# Association of lamina cribrosa thickness and hippocampal volume in Alzheimer's disease patients

**DOI:** 10.1055/s-0044-1791658

**Published:** 2024-11-03

**Authors:** Ersin Kasım Ulusoy, Döndü Melek Ulusoy, Mehmet Fatih Göl, Ayşe Çiçek, Turgut Tursem Tokmak

**Affiliations:** 1 City Hospital of Ankara, Neurology Department, Ankara, Turkey.; 2 City Hospital of Ankara, Ophthalmology Department, Ankara, Turkey.; 3 City Hospital of Kayseri, Neurology Department, Kayseri, Turkey.; 4 City Hospital of Kayseri, Ophtalmology Department, Kayseri Turkey.; 5 City Hospital of Kayseri, Radiology Department, Kayseri, Turkey.

**Keywords:** Alzheimer Disease, Hippocampal Volume, Lamina Cribrosa Thickness, Doença de Alzheimer, Volume do Hipocampo, Espessura da Lâmina Cribrosa

## Abstract

**Background** Alzheimer's disease (AD) is the most common cause of dementia and affects a large portion of the elderly population worldwide.

**Objective** To analyze the relationship between lamina cribrosa thickness (LCT) and hippocampal volume in patients with AD and mild cognitive impairment (MCI).

**Methods** The sample in the present study consisted of 20 recently diagnosed MCI patients, 20 recently diagnosed AD patients, and 20 matched healthy volunteers. Every patient underwent magnetic resonance imaging (MRI) scans. The VolBrain software (open-access platform for MRI brain analysis) was used to calculate the hippocampal volume. Optical coherence tomography was performed to measure the LCT. Analysis of variance and Pearson chi-squared tests were employed to assess the results.

**Results** The lowest total hippocampal volume ( *p* < 0.05) was in the AD group, which was 6.14 ± 0.66 mm ^3^ , while in the control group, it was 7.7 ± 9.65 mm ^3^ , and 6.69 ± 0.46 mm ^3^ in the MCI group. In comparison to the rest of the groups, in the AD group, the LCT was the thinnest (202.17 ± 16.35 µm). As per the results of the study population as a whole, low hippocampal volume causes low LCT, which shows an important relationship (r: 0.41; *p* < 0.05).

**Conclusion** The current findings present evidence of the relationship between hippocampal volume and LCT in patients with AD and MCI.

## Resumo

**Antecedentes** A doença de Alzheimer (DA) é a causa mais comum de demência e afeta uma grande parcela da população idosa em todo o mundo.

**Objetivo** Analisar a relação entre a espessura da lâmina cribrosa (ELC) e o volume do hipocampo em pacientes com DA e comprometimento cognitivo leve (CCL).

**Métodos** A amostra neste estudo consistiu em 20 pacientes com diagnóstico recente de CCL, 20 pacientes com DA recentemente diagnosticados e 20 voluntários saudáveis pareados. Todos os pacientes foram submetidos a exames de ressonância magnética (RM). O programa VolBrain (plataforma de acesso aberto para análise cerebral por RM) foi utilizado para calcular o volume do hipocampo. Tomografia de corrência óptica foi feita para medir a ELC. Os testes análise de variância (ANOVA) e qui-quadrado de Pearson foram empregados para avaliar os resultados.

**Resultados** O menor volume total do hipocampo ( *p* < 0,05) foi no grupo DA, que foi 6,14 ± 0,66 mm ^3^ , enquanto no grupo controle foi 7,7 ± 9,65 mm ^3^ e 6,69 ± 0,46 mm ^3^ no grupo CCL. Em comparação com os demais grupos, no grupo DA, a ELC foi a mais fina (202,17 ± 16,35 µm). De acordo com os resultados da população estudada como um todo, o baixo volume do hipocampo causa baixa ELC, o que mostra uma relação importante (r: 0,41; *p* < 0,05).

**Conclusão** Os presentes achados apresentam evidências da relação entre o volume do hipocampo e a ELC em pacientes com DA e CCL.

## INTRODUCTION

Alzheimer's disease (AD) is a neurodegenerative disorder of the central nervous system (CNS) with an occurrence rate of approximately 10% in the older age group. 1 2 The neuropathological markers for AD are amyloid beta (Aβ) plaques and tau-containing neurofibrillary tangles (NFT). 3 4 5 The AD diagnosis is made by clinical criteria together with specific markers showing neuronal injury and AD pathology. Symptoms of neuronal damage include hypometabolism on fluorodeoxyglucose-PET (FDG-PET), elevated tau and/or phosphorylated tau (pTau) levels in cerebrospinal fluid (CSF), or cortical atrophy on magnetic resonance imaging (MRI). Low levels of Aβ in CSF or on an amyloid positron emission tomography (PET) determines Aβ pathology 6 7 . However, the investigation of these markers is costly, invasive, and time-consuming. Hence, there is urgently need to have an AD biomarker that is basic, cost-effective, patient-friendly and, preferably, that helps with early AD pathology diagnosis of neurodegeneration in its initial phase.

The optic nerve is embryologically derived from the neural tube, and as a protrusion from the brain, it shares many similarities with brain tissue. 8 Primarily, axons of retinal ganglion cells (RGCs) extend to the optic chiasm and tract and create synapses at the lateral geniculate nucleus eventually forming the optic nerve. 9 10 Therefore, in the process of neurodegeneration, the optic nerve may also be impacted in the same way. It is deduced that the pathogenic mechanisms that take place in retinal degenerative diseases, such as AD and Parkinson disease (PD), are similar to those of neurodegenerative diseases. 10

The major area of axonal damage is the lamina cribrosa (LC), which is a mesh-like structure that borders and safeguards the retinal ganglion cells (RGCs) axons at the optic nerve head (ONH). The optic nerve support at the LC level is reduced and becomes more vulnerable to damage due to such structural changes. 11 Thus, to analyze the acuteness and the time frame of neurodegenerative disease, it is crucial to analyze the optic nerve head. Vivo imaging of the LC can be obtained using optical coherence tomography (OCT). Recent advances in OCT have made it possible to observe in-vivo the deep structures of the ONH and, in particular, the LC. Optical coherence tomography is a non-contact, high-resolution, in-vivo imaging modality and widely adopted for clinical care to objectively assess small-scale changes in the eye. 12

The analysis of hippocampal volume and the thickness and depth of the LC in patients with early-onset AD and MCI and the comparison between age-matched cognitively normal healthy subjects, using quantitative volumetric MRI analysis with the VolBrain software (open-access platform for MRI brain analysis), are the goals of the present study.

## METHODS

### Study population and design

The present cross-sectional study was conducted at the Neurology, Radiology, and Ophthalmology Department of Kayseri City Hospital, in Kayseri, Turkey, between April 2022- December 2023. The regional ethics committee approved the study, which abided by the laws of the Declaration of Helsinki. After the participants got oral and written information regarding the study, they provided written consent.

### Inclusion criteria

Twenty AD and 20 MCI patients were a part of our study and were compared with 20 age-, sex- and schooling-level-matched healthy volunteers. They were admitted to a neurology outpatient clinic and diagnosed according to classification standards of the National Institute on Aging-Alzheimer Association. 13 The individuals reporting no cognitive complaint or systemic, psychiatric, or neurological disorder which may initiate neurodegenerative and neuropsychiatric disease were used as a control group.

Age, sex, and schooling level were recorded for every participant. Additionally, the Mini-Mental State Examination (MMSE) was used to evaluate their cognitive function. According to the results of the MMSE, early dementia was diagnosed for the patients scoring 17 to 23 points, and MCI for those who scored 24 to 30 points. 4

Magnetic resonance imaging and OCT scan were done on every subject.

### Exclusion criteria

The patients not included were those with neurodegenerative (like multiple sclerosis, PD) or neuropsychiatric issues, patients younger than 65 or older than 75, patients with a score < 17 points in the MMSE, patients with repeated head injury or CNS infection within the 5 years prior to study recruitment, those with moderate-to-severe white or grey matter lesions, patients who previously had intracranial surgery or tumor, and those with a history of ischemic or hemorrhagic stroke.

Additional ophthalmological exclusion criteria were as follows: corneal pathology, dense media opacities, history of uveitis, glaucoma, an abnormal appearing optic disc without glaucomatous optic neuropathy and pallor or swelling, axial length of more than 26 mm, ocular trauma, and previous intraocular surgery. Moreover, patients with history of systemic disease, such as hypertension or diabetes mellitus, or patients who were using any medication were also excluded.

### Neuroimaging (web-based brain volume calculation)

Neuroimaging was done using the 1.5 T Siemens Aera MR scanner (Siemens AG, Berlin, Germany). Structural images were taken by using T1-weighted 3D magnetization. In the sagittal plane, a magnetization-prepared rapid gradient-echo (MPRAGE) sequence was prepared by using acquisition matrix = 256 × 256, TE/TR = 1,900 ms/2.84s, FOV = 280 mm ^2^ , number of slices = 160, slice thickness = 1.0 mm, and flip angle = 5 ^o^ .

The Picture Archiving and Communication System (PACS) server was employed to download the T1 MRI data, which was then processed and transferred using another software. To assess the volume, MRI data was converted to the Neuroimaging Informatics Technology Initiative (NIFTII) format. To do this, a 32-bit Dell PC (Dell Technologies Inc., Round Rock, TX, USA), with Micosoft Windows 10 operating system (Microsoft Corp., Redmond, WA, USA), was used. We calculated the volume by using volBrain. There is no need for configuration, installation, or training to use the volBrain pipeline. A web interface employing a software as a service (SaaS) model to automatically generate a report, which consists of volumetric information from any submitted case, is the medium for the volBrain volumetric analysis system to work remotely. 14 15

### Eye examination protocol and study measurements

Every patient underwent a comprehensive ophthalmic examination that included measuring intraocular pressure (OP) using Goldmann applanation tonometry, assessing visual acuity, looking for signs of spherical and cylindrical refractive errors, fundus examination, slit lamp biomicroscopy, gonioscopy, axial length measurements (IOL Master; Carl Zeiss Meditec, Dublin, CA, USA), measuring central corneal thickness using a Scheimpflug camera (Pentacam; Oculus Optikgeräte GmbH, Wetzlar, Germany), and scanning the optic disc using enhanced depth imaging (EDI) spectral domain OCT (SD-OCT) (Spectralis OCT; Heidelberg Engineering, Heidelberg, Germany). The tests above were done on every patient.

### Spectral domain OCT

Using the cyclopentolate, the pupils were completely dilated. Following pupillary dilation, the LCT of patients and controls was measured using images captured using a Heidelberg Spectralis SD-OCT imaging device with an EDI program. The analysis did not include the scan results with quality scores of 20 and scans with low quality classified by the vague images of the fundus or a vague boundary of the LCT.

### Enhanced depth-imaging SD-OCT of the optic nerve head

**Figure 1:**
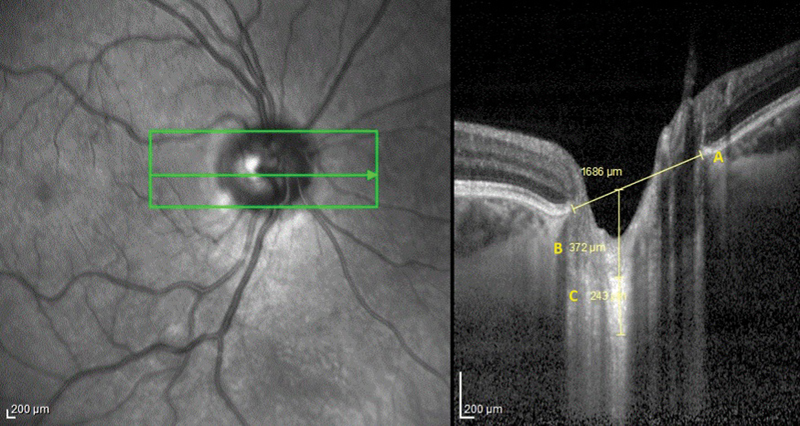
Optic nerve head's enhanced depth imaging optical coherence tomography B-scan. **A.** Bruch membrane opening (BMO); **B.** Lamina cribrosa depth (LCD); **C.** Lamina cribrosa thickness (LCT).

The technique was published for the EDI of the optic nerve head (ONH) with SD-OCT. 16 In short, the SD-OCT equipment captured a 15 × 10-degree rectangle centered on the optic disc. Sixty-five individual pieces formed this rectangle, in which an average of 100 OCT frames were present in every sector. Three frames were selected by us (center, mid superior, and mid inferior) from these horizontal B-scans that were transmitted through the ONH. The parameters in each of these frames were measured. To measure thickness, we positioned the weight of every LCT plate in the center. The Bruch membrane opening (BMO) and LCT borders in the ONH OCT images of an AD patient and a control are illustrated in Figure 1 . The line that joins two ends of the Bruch membrane is called the BMO.

Measurement for distances was taken on the line perpendicular to the reference line. As much as it was possible, the measurements were acquired very closely to the vertical center of the ONH. When the measurements were struck by a vascular trunk, the temporal side was utilized to measure. The highly reflecting region's anterior and posterior bounds at the vertical center of the ONH served as the LCT borders in the horizontal SD-OCT cross-section, and the LCT was defined as the area between them.

To locate images that clearly displayed the LCT, the contrast settings were changed. The distance between the anterior edge of the LCT and the BMO was known as the lamina cribrosa depth (LCD). The HEYEX software, version 6.0, (Heidelberg Engineering Inc.) was used to acquire the measurements. One examiner (MU) conducted each measurement three times, using the average of the three values as the primary basis for analysis.

### Statistical analysis

The IBM SPSS Statistics for Windows version 20.0 (IBM Corp., Armonk, NY, USA) was used to perform every evaluation. The numerical variables were expressed as mean ± standard deviation values, while the qualitative variables, as numbers and percentages. The normality assessment was done using the Kolmogorov-Smirnov test. To compare the groups for continuous variables, a one-way analysis of variance (ANOVA) for parameters with normal distribution was employed, and for parameters that do not have normal distribution, the Kruskal-Vallis test was performed. For parametric and nonparametric comparisons, the Pearson Chi-squared and Fisher exact tests were performed, respectively, for categorical variables. To assess the relationship between the hippocampus volume and the LCT, the Pearson correlation coefficient was calculated.

## RESULTS

There were 20 AD and 20 MCI patients and 20 healthy controls in the study. All patients' eyes were examined. Table 1 shows a contrast of the primary clinical characteristics between the AD, MCI, and healthy groups. Other primary characteristics, such as gender, age, IOP, schooling, axial length, and central corneal thickness did not show any major difference between the AD, MCI, and healthy groups ( Table 1 ). The mean MMSE score was 26.57 ± 1.4 in MCI patients and 20.48 ± 2.31 in AD patients.

**Table 1:** Demographics of the study sample

	Control group (n = 20)	MCI group (n = 20)	AD group (n = 20)	*p* -value
Age(years)	70 ± 3.56	70.35 ± 3.72	71 ± 3.6	0.39
Gender (male; female)	8; 12 (40%; 60%)	8; 12 (40%; 60%)	7; 13 (35%; 65%)	0.72
Education (illiterate; primary; secondary; high school)	6; 10; 2; 2 (30%; 50%; 10%; 10%)	7; 11; 2 (35%; 55%; 10%)	9; 10; 1 (45%; 50%; 5%)	0.11
MMSE	−	26.57 ± 1.4	20.48 ± 2.31	0.76
Axial length (mm)	23.42 ± 0.6	23.55 ± 0.7	23.57 ± 0.61	0.51
IOP (mmHg)	14.35 ± 2.3	14.5 ± 1.85	14.47 ± 2.2	0.94
Central corneal thickness (µm)	524.4 ± 29.35	535.15 ± 33.23	529.37 ± 25.56	0.27

Abbreviations: AD, Alzheimer's disease; IOP, intraocular pressure; MCI, mild cognitive impairment; MMSE, Mini Mental State Examination.

In the control group, the total hippocampal volume was 7.7 ± 9.65 mm ^3^ , while in the MCI group, it was 6.69 ± 0.46 mm ^3^ , and the lowest one was in the AD group ( *p* < 0.05), with 6.14 ± 0.66 mm ^3^ . These groups ( *p* < 0.05) showed considerable differences between them, with the biggest difference being seen in the AD group, in which the individual examination revealed major differences for the right and left hippocampal volumes ( *p* < 0.05; Table 2 ).

**Table 2:** Hippocampal volumes among the study sample

	Control group (n = 20)	MCI group (n = 20)	AD group (n = 20)	*p* -value
Hippocampus (total) (mm ^3^ )	7.7 ± 0.65	6.69 ± 0.46	6.14 ± 0.66	0.001
Hippocampus (right) (mm ^3^ )	3.87 ± 0.30	3.38 ± 0.23	3.01 ± 0.35	0.001
Hippocampus (left) (mm ^3^ )	3.81 ± 0.40	3.42 ± 0.24	2.97 ± 0.67	0.001

Abbreviations: AD, Alzheimer's disease; MCI, mild cognitive impairment.

The LCT and LCD between the AD, MCI, and healthy groups were compared in Table 3 . The mean LCTs were 202.17 ± 16.35 µm, 229 ± 16.66 µm, and 246.37 ± 11.11 µm, respectively. In the AD and MCI group, the LC was noticeably thinner when compared to the healthy group ( *p* < 0.001). The mean LCDs were 435.55 ± 99.9 µm, 436.22 ± 83.03 µm, and 433.6 ± 57.67 µm for the AD, MCI, and control groups, respectively ( *p* = 0.76), which did not represent a major difference.

**Table 3:** Comparison of lamina cribrosa thickness and depth among the study sample

	Control group (n = 40)	MCI group (n = 40)	AD group (n = 40)	*p* -value
LCT (µm)	246.37 ± 11.11	229 ± 16.66	202.17 ± 16.35	0.001
LCD (µm)	433.6 ± 57.67	436.22 ± 83.03	435.55 ± 99.9	0.76

Abbreviations: AD, Alzheimer's disease; LCD lamina cribrosa depth; LCT, lamina cribrosa thickness; MCI, mild cognitive impairment.

According to the results of the entire study population, low hippocampal volumes correlated with reduced LCT; therefore, they share a crucial relationship (r = 0.41; *p* < 0.05). In the AD group, this correlation was moderate, while it was seen as stronger in the MCI group ( *p* < 0.05; Table 4 ).

**Table 4:** Correlations between lamina cribrosa thickness and hippocampus volume among the study sample

	Control group	MCI group	AD group
**Pearson correlation coefficient (r)**	−	0.79	0.41
***p*** **-value**	0.68	< 0.001	0.007

Abbreviations: AD, Alzheimer's disease; LCT, lamina cribrosa thickness; MCI, mild cognitive impairment.

## DISCUSSION

The correlation between hippocampal volume and LCT among patients with AD, MCI, and controls with different ages, sexes, and schooling levels was compared in this study. According to our results, when compared to the controls, a decrease in both hippocampal volume and LCT in the early AD group was observed. Furthermore, a relationship between hippocampal volume and thinning in the LC was seen in our study. As far as we know, the relationship between hippocampal volume and LCT in AD has been studied for the first time by us.

The accumulation of Aß plaques and NFTs in certain regions of the brain results in AD, which is a neurodegenerative disorder. As per the literature, the transentorhinal and entorhinal cortexes are the starting points for the neuropathology, which then extends to the hippocampus affecting the neocortex and limbic system, eventually. It is suggested that a pathological correlative for the continuum of normal ageing-MCI-dementia is the NFTs and Aß plaques being collected in the limbic system and progressing to the neocortex. The studies on particular brain regions, such as the hippocampus, corpus callosum, cerebellum, and frontal and temporal lobes, show that in the context of early diagnosis of AD, these areas are crucial. 1 17 In light of these data, we conducted a study on the hippocampus in order to demonstrate AD diagnosis.

The hippocampus is situated within the limbic system, which is crucial in regulating movements and behaviors. It is also responsible for learning, spatial navigation, and memory. The hippocampus was seen as relatively preserved in neurodegenerative disorders, except in AD, in which it is one of the preliminary regions involved, according to existing studies. 18 In a study on patients at risk for familial AD, Fox et al. aimed to determine the earliest time for the onset of hippocampal changes and showed that hippocampal atrophy started during the asymptomatic period. 19 Saeed et al. investigated the association between hippocampal volume and the apolipoprotein E (APOE) genotype, reporting a significant correlation. 20 In our study, we performed volumetric calculation of the hippocampus in patients with AD and MCI and compared the results with those obtained in healthy volunteers. Our data showed that hippocampal volume was lower in the AD group when compared with the MCI and control groups.

Alzheimer's disease is the most commonly seen neurodegenerative disorder, comprising the most common cause of dementia in the elderly population. Ancillary techniques may occasionally be necessary to distinguish AD from other types of dementia when clinical diagnostic criteria are not sufficient. 21 Studies that use structural changes in the optic disc to identify and quantify the amount of amyloid deposition in the CNS are becoming more and more common. Postmortem examinations of the globe revealed a link between the severity of the disease and tau, Aß plaques, ubiquitin, and α-synuclein. 22 Unfortunately, due to their high cost, intrusive nature, and lengthy processing time, these markers could not be implemented into clinical practice. As a result, non-invasive techniques for identifying retinal alterations as a sign of neurodegeneration have drawn attention and shown promise. 23 In this study, we assessed disease progression by measuring LCT via non-invasive OCT scanning.

The area through which optic nerve fibers pass and connect to upper centers is known as the LC. The LC consists of extracellular matrix, astrocytes, and microglial cells. It is believed that LC supports the axons of retinal ganglion cells, both structurally and metabolically. 24 25 There has been a suggestion that RGC axons are more vulnerable to mechanical harm. An increasing amount of data suggests that the laminar region of the ONH sustains injury prior to axonal loss. It is unclear, therefore, if pathological or physiological aging is the cause of LC thinning. It has been suggested that elevated tau protein accelerates the process of neurodegeneration, making laminar astrocytes more vulnerable to degeneration. 26 27 In an AD investigation, Lee et al. discovered that patients with greater CSF tau protein levels had thinner LC. 28 In a study using OCT, Kayabaşi et al. injected Aß-binding protein in patients with MCI and found more inclusion cells in the retina when compared to healthy controls. 29 We expanded these findings by studying and comparing hippocampal volume in patients with AD and MCI. In our study, it was found that the LC was thinner in patients with low hippocampal volume. We think that this could be useful in illustrating the connection between retinal and brain cell losses.

In recent years, advanced neuroimaging techniques have made it possible to quantify the volumetric composition of many cerebral structures, which is now utilized to diagnose and track the evolution of certain illnesses. 30 31 We can perform novel studies on the anatomy and functions of the brain by combining MRI with software that calculates brain volumes. 32 33 In a study by Sheelakumari et al., 4 volumetric measurements were performed at cerebral structures such as temporal lobe, hippocampus, amygdale, and thalamus, and results were compared between patients with AD and controls in a clinico-volumetric manner. However, the authors 4 did not correlate these results with other biomarkers. After analyzing the data, we measured the hippocampal volume in this study and compared the results with LCT, which may be a possible prognostic/diagnostic. Our results indicated a correlation between hippocampal volume loss and LCT.

In terms of volumetric measurement, the hippocampus is a more dependable structure than other structures to predict the progression of MCI to AD. It is estimated that 12% of MCI patients annually have a chance of developing AD, and that by the time they are 6 years old, roughly 80% will have dementia. 34 In order to stop the disease from progressing and to enhance the patient's quality of life, it is crucial to start using a disease-modifying medication early on. Hippocampal atrophy has reportedly been identified in earlier research as a potential marker for the transition of MCI to AD. Haan et al. investigated the correlation between retinal thinning and cortical volume in patients with AD and found a correlation between retinal thinning and parietal lobe atrophy. 8 The correlation between AD and LC thinning was investigated in a few studies. However, none showed a correlation between LC thinning and hippocampal volume in AD. Our study is the first showing such a correlation.

The present study has some limitations, including small sample size and cross-sectional design. It is further restricted by the fact that the diagnosis of AD was based solely on clinical and MRI symptoms, rather than on the results of specialized testing that would have revealed amyloid deposition, like positron emission tomography (PET) or CSF studies. Thus, we may have included patients with different types of dementia. Another limitation is that we measured the patients' cognitive status only with MMSE, without using any other neurocognitive test. In addition, more comprehensive studies and postmortem histopathological investigations are needed to validate this hypothesis.

In conclusion, studies on non-invasive assessments for early-stage AD and MCI patient diagnosis and progression are becoming more numerous. It is valuable to assess the optic disc, which is considered as a continuum of the brain and has several similarities with it. In previous studies using different methods, it was reported that the LC and AD showed pathophysiological involvement. To the best of our knowledge, nevertheless, this is the first study showing a connection between LCT and hippocampus volume in AD and MCI patients. In addition, we suggest that assessment of the LC immediately after onset on pathological process via non-invasive methods can allow early diagnosis and facilitate preventive measures against disease progression.
